# Immune checkpoints in MSI and CSI colorectal cancers and their translational implications

**DOI:** 10.1186/2051-1426-1-S1-P161

**Published:** 2013-11-07

**Authors:** Nicolás J Llosa, Franck Housseau, Elizabeth Wick, Lizzy Hechenbleikner, Michael Cruise, Robert Anders, Cynthia Sears, Drew Pardoll

**Affiliations:** 1 Oncology, Johns Hopkins Hospital, Baltimore, MD, USA

## Background

Cancer vaccines attracted a lot of attention as an alternative/adjuvant therapeutic approach for patients with advanced metastatic cancer. Preclinical studies showed that tumors can evade the adaptive immune response by activation of immune checkpoints in the tumor micro-environment. Tumors may naturally up-regulate immunosuppressive ligands in response to the host immunity, making these immune checkpoints critical components of a tumor-associated immune signatures and useful prognostic biomarkers since their immunosuppressive function can be reversed by specific and drugable inhibitors. We postulate that the mechanism(s) of resistance to adaptive immunity may be distinct between the genetically distinct MSI+ and CSI+ CRC.

## Results

Thirty three tumor/normal pairs, including 7 MSIhigh samples have been analyzed for their immunologic environment, including lymphocytes subsets, cytokine production, immune checkpoint expression and myeloid populations. Flow cytometry analysis showed Lag-3 or Tim-3 predominant patients in CD4+ and CD8+ cells and these two immune-checkpoints behave in a mutually exclusive pattern. CD4+ cells made higher amounts of IL-17 in MSIlow patients compared to the prognostically better MSIhigh patients group. Conversely, the expression of Tim-3 was higher on CD4+ cells from MSIhigh patients suggesting a more active state of these lymphocytes. In these two genetically distinct CRC populations, immune checkpoints seem to have a different functional significance. In MSIlow samples, there is an increased production of interferon gamma in tumor-infiltrating T cells (CD4+ and CD8+) versus a decreased production of this cytokine in cells expressing LAG-3 or TIM-3. On the contrary, while observing a very strong production of IFNγ in MSIhigh CRC-infiltrating T cells, LAG-3+ or Tim3+ T cells barely decreased IFNγ production. Finally, according to the PD-1 expression of CD4+ cells in MSIhigh patients, we identified two distinct populations of lymphocytes which react differently to Lag-3 or Tim-3 expression. A PD-1 high population, which is a stronger producer of IFNγ and is not inhibited by Lag-3 or Tim-3 expression except in the presence of high levels of Lag-3, and a PD-1 low CD4 population which produces much lower quantities of IFNγ which is suppressed by the presence of the two mentioned immune-checkpoints.

## Conclusions

Our data has direct translational implications for the use of the MSI vs CSI CRC status to design clinical trials with relevant immunomodulatory mAbs. Patients with low expression of PD-1 may therefore be more sensitive to the LAG-3 or TIM-3 blockade approach.

